# Effectiveness of non-invasive ultrasound-guided electrical stimulation of genicular nerves for chronic knee pain: A case report

**DOI:** 10.1097/MD.0000000000041286

**Published:** 2025-01-17

**Authors:** Futoshi Ikuta, Masashi Matsuzaki, Kotaro Kajitani

**Affiliations:** a School of Health Sciences, Tokyo International University, Kawagoe, Saitama, Japan; b Sonic Japan Holdings Co., Ltd, Hachioji, Tokyo, Japan; c Uenohara Kajitani Orthopaedics, Uenohara, Yamanashi, Japan.

**Keywords:** electrical nerve reactivation, ENR, chronic knee pain, ultrasound-guided, genicular nerve, Osteoarthritis, case report

## Abstract

**Rationale:**

Chronic knee pain is a common health issue that requires effective and noninvasive treatment. We devised a novel noninvasive approach using ultrasound-guided electrical nerve reactivation (ENR) in which ultrasound is used to identify the genicular nerve (GN). Then, transcutaneous low-frequency stimulation is applied for 10 seconds. The aim of this study was to clarify the pain-relieving effects of ENR on the GN innervating the knee joint.

**Patient concerns:**

Patients had visited our hospital with the complaint of knee joint pain.

**Diagnoses:**

This study included 21 osteoarthritic knees from 16 patients with a mean age of 78.3 ± 8.5 years.

**Interventions:**

Baseline measurements included the range of motion of the knee joint, Visual Analogue Scale (VAS) scores for pain, and maximum knee flexion angle during squatting. A therapist conducted an interview to locate the painful area of the knee and then performed ultrasound-guided ENR by targeting the GN.

**Outcomes:**

The preintervention VAS score was 49.3 (95% confidence interval: 41.0, 57.5). Postintervention VAS scores showed significant reductions: 27.0 (19.0, 35.1) immediately after the intervention (*P* < .0001), 27.7 (22.6, 32.8) at 1-day postintervention (*P* = .002), and 29.0 (22.9, 35.1) at 1-week postintervention (*P* = .001). The knee flexion angle during squatting significantly improved from 108.1° (101.6, 114.5) preintervention to 121.9° (115.1, 128.7) postintervention (*P* < .001). There was a significant increase in flexion angle preintervention and postintervention (*P* = .02); however, no significant change was observed in the extension angle. In addition, no adverse events were reported.

**Lessons:**

This study is the first to demonstrate the effects of ultrasound-guided low-frequency stimulation of the GN for knee osteoarthritis. Although the exact mechanism of pain relief is unclear, we hypothesize that alterations in neurotransmission, with or without endorphin release, may play a role. Moreover, ENR may improve nerve entrapment by causing contraction of surrounding muscles.

This study demonstrated that ultrasound-guided ENR targets the GN and effectively reduces pain without complications. This treatment addresses the limitations of invasive methods, such as hydrorelease and radiofrequency ablation. Furthermore, ultrasound-guided ENR has diverse applications in diagnostics, physical therapy, and pre-exercise pain management and will greatly benefit patients and healthcare professionals.

## 1. Introduction

Chronic pain is a common, complex, and distressing problem that significantly impacts both society and individuals.^[1]^ Recently, ultrasound-guided injection techniques have gained popularity as a treatment for chronic pain.^[2]^ This method, known as hydrodissection or hydrorelease, involves using an anesthetic or solution such as saline to separate the nerve from the surrounding tissue, fascia, or adjacent structures.^[3]^ Hydrorelease also employs ultrasound-guided injection but has a broader range of indications beyond just nerves.^[4,5]^ Ultrasound-guided injections offer many benefits for patients, including pain relief and improved sensation.^[6]^

Ultrasound-guided injection is not without problems. An unavoidable concern is the fear and pain it causes patients. Risks such as nerve damage^[7]^ and infection must also be considered. In clinical settings, the time and effort required to prepare injections and obtain informed consent from patients burdens healthcare providers. Additionally, it can be challenging to obtain patient consent to inject another site if the first injection was ineffective. To address these issues, we have devised a non-invasive ultrasound-guided electrical nerve reactivation (ENR) that is an ultrasound-guided treatment applying electrical stimulation to the nerve triggering the pain. A literature review on the mechanism of peripheral nerve stimulation in chronic pain has demonstrated that this stimulation modulates inflammatory pathways, the autonomic nervous system, and endogenous pain inhibition pathways, involving cortical and subcortical areas.^[8]^ Therefore, ultrasound-guided ENR is expected to efficiently reduce pain and, in turn, increase muscle output.

Osteoarthritis (OA) is one of the leading causes of chronic pain, with knee OA being highly prevalent.^[9]^ This condition poses significant problems not only for patients but also as a major economic issue.^[10]^ The effectiveness of radiofrequency neurotomy on the genicular nerve (GN) for chronic pain in knee OA has recently been reported.^[11,12]^ This procedure uses the heat produced by radiofrequency waves to denature proteins in the nerve sheaths of the GN, thereby “switching off” pain transmission.^[12]^ While effective, this treatment is invasive and can be difficult for patients to endure. We propose that ENR is effective for knee pain caused by GNs.

This study aimed to clarify the pain relieving effect and duration of ENR. Our hypotheses were that “ENR reduces knee joint pain and increases the knee flexion angle during squatting” and that “the pain reduction effect of ENR is temporary and returns the next day.”

## 2. Methods

This study compared pre- and post-intervention outcomes without a control group. This study included patients with knee OA who regularly attended our hospital. In all cases, the diagnosis of knee OA was made by the same experienced doctor. Inclusion criteria were patients with confirmed tenderness in the GNs as identified by ultrasound. Patients who were informed about the treatment but did not agree to participate were excluded.

The study protocol was approved by the ethics committees of our institutions and conducted in accordance with the tenets of the Declaration of Helsinki. All patients gave written informed consent before the treatment. The patients were shown the device to explain that the treatment equipment is safe to use at home as a low-frequency therapy device. The patients were then informed of the expected benefits of this treatment and that some pain would occur during treatment. All participants provided consent for the publication of this case report.

**Figure 1.**
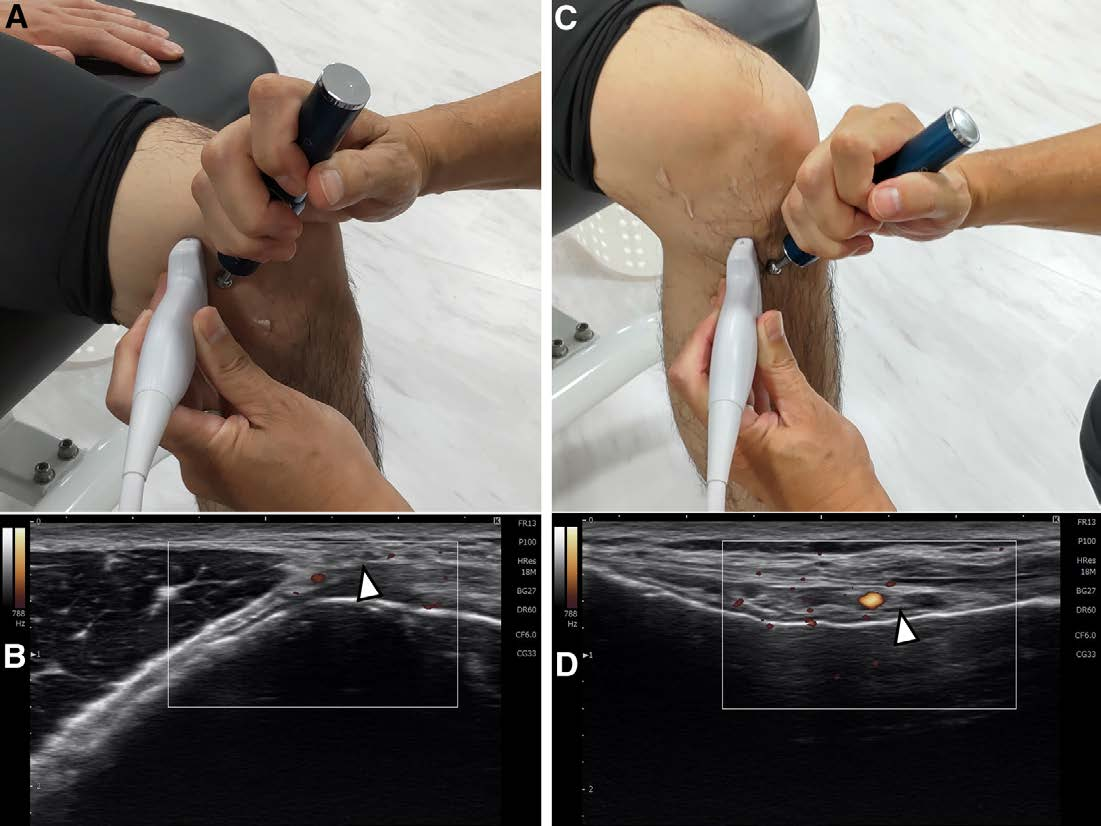
Genicular nerves are difficult to detect on ultrasound images, so arteries are used as an indicator. (A, B) The transducer placement is indicated for the cross-sectional view of the left superior medial genicular artery with a power Doppler image (white arrow) and (C, D) the left inferior medial genicular artery with a power Doppler image (white arrow).

Before the intervention, the patient’s knee range of motion, Visual Analogue Scale (VAS) score, and maximum knee flexion angle during squatting were measured. Squat was performed naturally, with the patient lightly touching a table to avoid falling. The patients performed a squat until they felt pain. Then, the examiner measured the flexion angle of the knee with a goniometer. ENR was then conducted in the following steps: the therapist interviewed the patient about the painful area of the knee and identified the dominant GN from that area.^[13]^ Specifically, vague pain in the upper medial side of the knee was suspected as superior medial GN (SMGN), the lower medial side as inferior medial GN (IMGN), the upper lateral side as superior lateral GN (SLGN), and the lower lateral side as inferior lateral GN (ILGN). Then, these 4 GNs were assessed for tenderness using ultrasound systems (SONIMAGE HS1, Konica Minolta Inc., Japan).^[12]^ Subsequently, electrical stimulation was applied to the tender GN for 10 seconds under ultrasound-guidance using a home-use, pen-type, low-frequency treatment device (Dr’Pen Pro, Setoworks Co., Ltd., China; Fig. 1). For patients with 2 tender points, low-frequency stimulation was applied at both locations. The same assessments conducted pre-intervention were repeated after low-frequency stimulation. Additionally, the VAS was assessed at the patient’s home the next day and 1 week later.

### 2.1. Statistical analysis

For each data set, a Shapiro–Wilk test was performed to check for normal distribution, followed by an analysis of variance. When a significant difference (*P* < .05) was observed in the analysis of variance, a multiple comparison method (corresponding *t* test or Wilcoxon signed rank test) was used. The multiple comparison method was set to α = .0083 (0.05/6) for the VAS, as it was measured 6 times in 4 groups (pre-intervention, post-intervention, 1 day, and 1 week). For the knee flexion angle during squatting and range of motion, α = .05 was used as these were comparisons between pre- and post-intervention. All statistical analyses were performed using EZR for Windows version 1.67.^[14]^

## 3. Results

**Figure 2.**
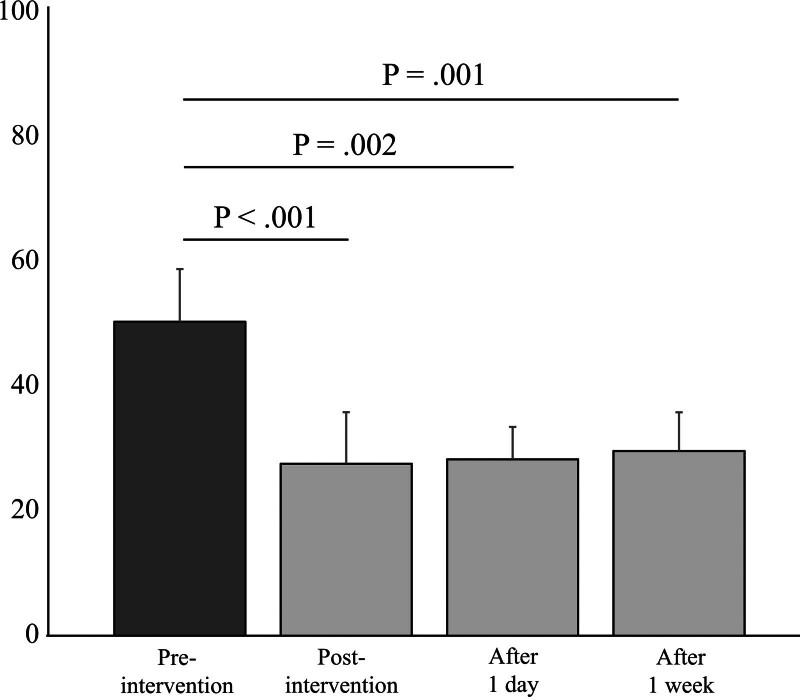
Visual analogue scale score among pre- and post-interventions. Error bars are 95% confidence interval.

Twenty-one knees of 16 patients (78.3 ± 8.5 years) were included in this study (Table 1). The Kellgren–Lawrence grades were as follows: 13 knees in grade 2, 6 knees in grade 3, and 2 knees in grade 4. The nerves treated were 11 SMGN, 2 SLGN, 2 IMGN, 2 ILGN, and 4 SMGN/IMGN. Pre-intervention VAS was 49.3 (95% confidence interval: 41.0, 57.5; Fig. 2). The VAS scores post-intervention were as follows: immediately post-intervention 27.0 (19.0, 35.1; *P* < .001), 1 day post-intervention 27.7 (22.6, 32.8; *P* = .002), and 1 week post-intervention 29.0 (22.9, 35.1; *P* = .001), all significantly different compared with pre-intervention. The flexion angle of the knee joint on the affected side during squatting significantly improved from 108.1° (101.6, 114.5) pre-intervention to 121.9° (115.1, 128.7) post-intervention (*P* < .001; Table 2). The flexion angle increased significantly from pre- to post-intervention (*P* = .02), but the extension angle did not differ significantly (Table 2). Moreover, no adverse events were reported.

**Table 1:** Demographic characteristics of the participants (n = 16).

Demographics characteristics	Number	%
Number of knees	21	
Sex		
Male	5	31.2
Female	11	68.8
K-L grade		
2	13	61.9
3	6	28.6
4	2	9.5
Demographics	Mean	Standard deviation
Age	78.3	8.5
Height (cm)	155.1	8.6
Weight (kg)	56.6	7.1
Body mass index (kg/m^2^)	23.5	1.9

**Table 2:** Range of motion of the knee (degrees).

	Pre-intervention		Post-intervention		*P* value
	Mean	95% CI	Mean	95% CI	
Extension	−6.2	−9.9, −2.5	−6.2	−9.9, −2.5	>.99
Flexion	133.8	129.2, 138.4	136.2	132.2, 140.1	**.02**
Squat	108.1	101.6, 114.5	121.9	115.1, 128.7	**<.001**

Bold values represent statistically significant (*P* < .05).

## 4. Discussion

This study is the first to report on the effects of using ultrasound to reliably apply low-frequency stimulation to the GN for knee OA. As a result, knee pain was reduced, and the flexion angle during squatting improved, without any adverse events.

The non-invasive ultrasound-guided ENR provided immediate pain relief. Electrical stimulation of nerves has been shown to reduce pain due to changes in endorphin release^[15]^ and neurotransmission.^[16]^ ENR not only stimulated the nerves but also caused contraction of the surrounding muscles. This muscle contraction may have increased blood perfusion and oxygen supply in ischemic tissues, reduced hyperalgesia in the peripheral and central nerves, and decreased involuntary muscle contractions.^[17]^ The contraction of muscles around the nerve may have improved nerve entrapment and reduced pain by a mechanism similar to hydrorelease.

The knee flexion angle during squatting was also improved by ultrasound-guided ENR. Since quadriceps muscle strength is important for squatting, it is believed that muscle output was enhanced by ENR.^[18]^ However, the GN is primarily a sensory nerve.^[19]^ Muscle contractions induced by electrical stimulation may have released the nerve and surrounding tissue, improving the nerve’s gliding properties, which in turn improved pain and flexion angle. Therefore, it is unclear whether ENR solely reduces pain and facilitates squatting, or if another mechanism is involved, which warrants future research.

Contrary to our hypotheses, the pain-relieving effects of ultrasound-guided ENR lasted for 1 week. There is evidence that transcutaneous electrical nerve stimulation for myofascial pain syndrome can improve pain and functional performance for up to 3 months.^[20]^ However, ENR took only 10 seconds to perform, which may indicate a different pain reduction mechanism than conventionally considered. Moreover, the temporary pain relief may improve knee function and maintain a pain-free state for a week. The pain-relief mechanism of hydrorelease also cannot be entirely explained by the release of nerve entrapment, suggesting the presence of overlapping mechanisms with ENR.

This study has several limitations. First, the mechanism of ENR is unclear. A typical mechanism for this is the gate control theory, which cannot be proven from this study.^[21]^ Second, a home electrotherapy machine was used, and its internal construction details are unknown. However, this suggests that even simple electrotherapy devices can provide ENR benefits. Finding optimal electric conditions must also be considered in the future. Third, we did not assess the patient’s muscle strength, administer questionnaires of physical function, or evaluate patient satisfaction. Although patients have a good reputation for ENR, future studies should investigate patient satisfaction. Fourth, this study did not have a control group. While placebo effects exist in transcutaneous electrostimulation,^[22]^ the brevity of ENR (only 10 seconds) and the patients prior experience with various treatments make it unlikely that a placebo effect dramatically reduced their pain. Finally, the extent of the ENR’s adaptation is unknown. The approach used in this study was effective against pain origination from GN; however, it is unclear whether the effective of our non-invasive approach will extend to knee pain of other causes. It is also unclear whether the approach would be effective against pain origination in other areas. However, we believe that the ENR is applicable to peripheral nerves throughout the body. Because the technique is unlikely to cause serious complications, we believe that it can be tried in practice and applied to the diagnosis of nerve pain and determination of treatment strategies. Nevertheless, there are many limitations to this study, and we present our findings as a case report.

However, the study has several notable strengths. First, as ultrasound-guided ENR is a non-invasive treatment, the risk of infection or nerve damage is virtually nonexistent. Second, patients feel safer and less fearful compared to hydrorelease. This is beneficial because if the first ENR is not effective, another area can be treated, increasing the likelihood of effectiveness. In clinical practice, we have observed similar effects to those in this study, not only in the sensory nerves but also in the peripheral nerves, and we intend to report these results in the future. Third, the burden on healthcare professionals is dramatically reduced compared to hydrorelease. Fourth, this technique can be useful for pain relief prior to physiotherapy or exercise. Finally, it can also reduce the financial burden on patients.

## 5. Conclusion

Ultrasound-guided ENR can reduce pain without complications. Furthermore, the technique has a wide range of potential uses, including diagnostic treatment, physiotherapy assessment, and pre-exercise pain relief.

## Acknowledgments

The authors would like to thank Dr Hiroshi Minagawa, the namer of hydrorelease, for providing useful discussions.

## Author contributions

**Conceptualization:** Futoshi Ikuta, Masashi Matsuzaki.

**Data curation:** Futoshi Ikuta, Kotaro Kajitani.

**Formal analysis:** Futoshi Ikuta.

**Investigation:** Futoshi Ikuta, Masashi Matsuzaki, Kotaro Kajitani.

**Methodology:** Futoshi Ikuta, Masashi Matsuzaki, Kotaro Kajitani.

**Project administration:** Futoshi Ikuta.

**Software:** Futoshi Ikuta.

**Supervision:** Futoshi Ikuta, Kotaro Kajitani.

**Validation:** Futoshi Ikuta.

**Visualization:** Futoshi Ikuta.

**Writing – original draft:** Futoshi Ikuta, Masashi Matsuzaki, Kotaro Kajitani.

**Writing – review & editing:** Futoshi Ikuta, Masashi Matsuzaki, Kotaro Kajitani.
